# Reactive Plasmacytosis Immediately After Immunosuppressive Therapy With Anti-human Thymocyte Immunoglobulin for Severe Aplastic Anemia: A Report of a Rare Case

**DOI:** 10.7759/cureus.57255

**Published:** 2024-03-30

**Authors:** Tomohito Shimada, Kana Bando, Atsushi Takahata, Shigeo Toyota

**Affiliations:** 1 Department of Hematology, Yokosuka Kyosai Hospital, Yokosuka, JPN

**Keywords:** reactive plasmacytosis, plasmacytosis, cyclosporine, anti-human thymocyte immunoglobulin, immunosuppressive therapy, aplastic anemia

## Abstract

Aplastic anemia is a hematopoietic deficiency disorder with pancytopenia, and immunosuppressive therapy is effective. We report a case in which plasma cells appeared in the peripheral blood during immunosuppressive therapy for aplastic anemia. Based on the results of morphology and flow cytometry, the plasma cells were considered reactive and disappeared spontaneously after follow-up. Thereafter, the patient had a good hematopoietic recovery. Reactive plasmacytosis has been reported in infectious and autoimmune diseases, but this is the first report of reactive plasmacytosis during immunosuppressive therapy for aplastic anemia, to our knowledge. In this case, reactive plasmacytosis was a sign preceding good hematopoietic recovery.

## Introduction

Aplastic anemia is a hematopoietic deficiency disease characterized by pancytopenia. Immunosuppressive therapy is effective because of the suppression of hematopoiesis by immunological mechanisms [1]. The current standard treatment for previously untreated severe aplastic anemia combines anti-human thymocyte immunoglobulin (ATG), cyclosporine, and eltrombopag [2]. ATG, in particular, is a key therapeutic drug. Because of its high T-cell suppressive activity, it has been used in the treatment of aplastic anemia since the 1970s and 1980s [3]. Currently, two types of ATG are available: horse ATG and rabbit ATG. However, it has been pointed out that the therapeutic efficacy of rabbit ATG may be inferior to that of horse ATG [4]. Horse ATG was initially used in Japan, but rabbit ATG has been used since 2008 after the discontinuation of its formulation. In March 2023, horse ATG (trade name ATGAM®) was approved and became available for use in Japan. This study reports a rare phenomenon of reactive plasma cells appearing in peripheral blood immediately after immunosuppressive therapy with ATGAM®. We discuss the relationship between aplastic anemia and plasmacytosis.

## Case presentation

A 56-year-old woman with no significant medical history presented to her primary care physician with a complaint of fatigue. Her blood tests revealed a marked pancytopenia with a WBC count of 3200/μL (neutrophils 752/μL, lymphocytes 2368/μL), Hb 3.5 g/dL, reticulocyte count 7470/μL, and platelet count of 7000/μL (Table 1). The patient was urgently admitted to our hospital for close examination and treatment.

**Table 1:** The patient’s laboratory findings on admission The patient had marked pancytopenia and serum iron and ferritin levels were significantly elevated. However, there were no other significant abnormal findings. MCV: mean corpuscular volume; MCH: mean corpuscular hemoglobin; MCHC: mean corpuscular hemoglobin concentration; μL: microliter; g/dL: gram per deciliter; fL: femtoliter; pg: picogram; mg/dL: microgram per deciliter; mEq/L; milli equivalent per liter; μg/dL: microgram per deciliter; ng/dL: nanogram per deciliter; pg/mL: picogram per microliter

Component and units	Lab value	Reference range
RBCs (10^6 cells/μL)	0.83 (Low)	3.76-5.00
Hemoglobin (g/dL)	3.5 (Low)	11.3-15.2
Hematocrit (%)	10.4 (Low)	33.4-44.9
MCV (fL)	125.7 (High)	79.0-100.0
MCH (pg)	42.6 (High)	26.3-34.3
MCHC (%)	33.9	30.7-36.6
Reticulocytes (‰)	9	2-26
White blood cells (cells/μL)	3,200 (Low)	3,500-9,100
Neutrophils (%)	23.5 (Low)	28.0-72.0
Lymphocytes (%)	74.0 (High)	18.0-58.0
Monocytes (%)	2.5	0.0-12.0
Eosinophils (%)	0.0	0.0-8.0
Basophils (%)	0.0	0.0-3.0
Neutrophils (cells/μL)	752 (Low)	1,500-6,500
Lymphocytes (cells/μL)	2,368	950-3,000
Platelets (cells/μL)	7,000 (Low)	130,000-369,000
Aspartate aminotransferase (U/L)	25	13-35
Alanine aminotransferase (U/L)	20	6-35
Lactate dehydrogenase (U/L)	224 (High)	124-222
Creatinine (mg/dL)	0.70	0.40-0.80
Blood urea nitrogen (mg/dL)	12	8-22
Sodium (mEq/L)	142	138-146
Potassium (mEq/L)	3.9	3.6-4.9
Chlorine (mEq/L)	105	99-109
Bilirrubin (mg/dL)	0.7	0.4-1.5
Albumine (g/dL)	4.0	4.0-5.0
Iron (μg/dL)	299 (High)	70-180
Total iron binding capacity (μg/dL)	332	290-355
Ferritin (ng/dL)	361 (High)	5-152
Folic acid (ng/mL)	8.8	3.9-12.9
Vitamin B12 (pg/mL)	448	233-914
C-reactive protein (mg/dL)	0.09	0.00-0.30

**Figure 1:**
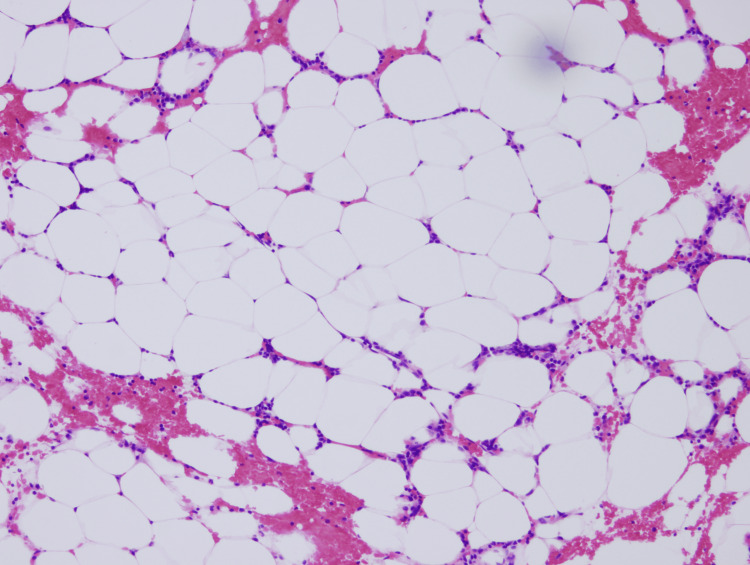
Bone marrow biopsy on admission (H&E stain) (×400） Bone marrow is hypocellular, with a majority of fatty tissue.

Bone marrow biopsy performed on the same day revealed a nucleated cell count of 5.5×10^4 /μL, megakaryocyte count below sensitivity, myeloid/erythroid (M/E) ratio of 0.64, and lymphocyte count of 24.4%. A bone marrow biopsy showed marked hypoplastic bone marrow, especially in the megakaryocytes, and no obvious malignant findings were observed (Figure 1). Peripheral blood paroxysmal nocturnal hemoglobinuria (PNH) hematology was positive for granulocytes at 18%. MRI of the thoracolumbar spine showed that the bone marrow was uniformly high-signal on T1-weighted images and low-signal on fat-suppressed images. Based on these findings, we diagnosed her with severe aplastic anemia.

**Figure 2:**
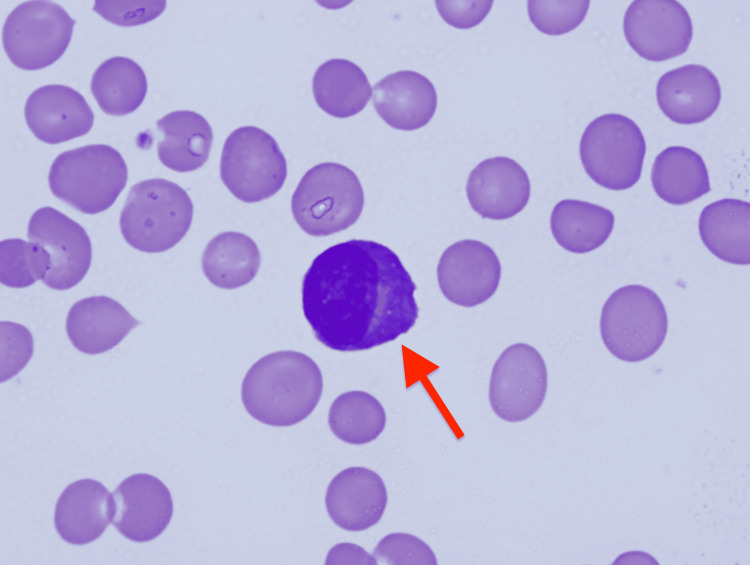
Plasma cell in peripheral blood smear (May-Giemsa stain) (×1000） The red arrow indicates a plasma cell in the peripheral blood.

**Figure 3:**
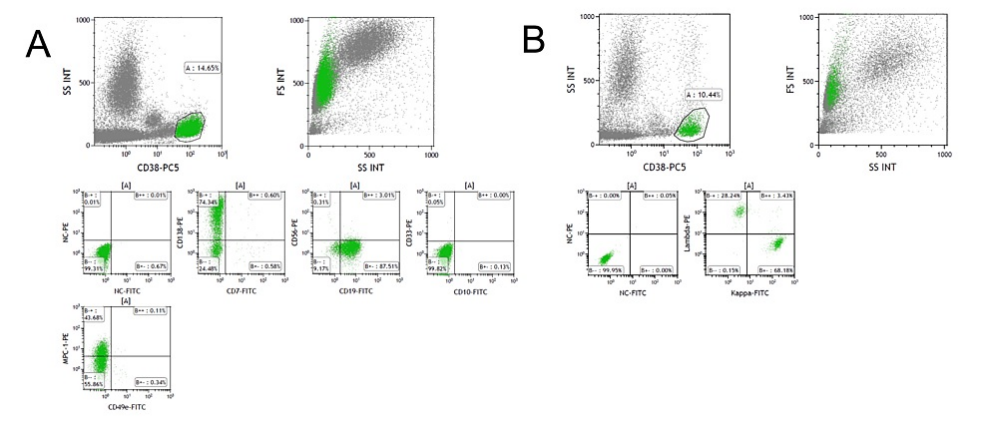
Peripheral blood flow cytometry findings A: CD38 gated flow cytometry of the peripheral blood identified plasma cells expressed CD19 and CD138. These plasma cells were negative for CD56. B: Also, these cells showed no deviation of cytoplasmic light chain expression. SS: side scatter; FS: forward scatter; INT: intensity

Because she was over 40 years old, we started immunosuppressive therapy with ATG, cyclosporine, and eltrombopag. We decided to start eltrombopag late because of concerns about its interaction with ATG. We started treatment with ATGAM® 40 mg/kg/day for four days and cyclosporine 5 mg/kg/day twice daily. Immediately after treatment, the patient developed no complications of note. However, on the 10th day of treatment, 18.5% of plasma cells (390 /μL) suddenly appeared in the peripheral blood (Figure 2). Morphologically, there was no evidence of dysplastic plasma cells such as binuclear or trinuclear cells. The patient had no symptoms, such as fever, lymphadenopathy, rash, and arthralgia. Blood tests showed that the inflammatory response and liver and renal functions were within normal limits. Peripheral blood samples were negative for Epstein-Barr virus (EBV)-DNA. Flow cytometric CD38 gating showed CD19-positive, CD56-negative, CD138-positive, and no light chain restriction (Figure 3), and no M protein was detected in the blood. Therefore, we concluded that the patient had reactive plasma cell proliferation without clonality. Treatment was continued under the policy of close observation. Plasma cell counts of approximately 10.0% (270 /μL) continued to appear in the peripheral blood until the 16th day of treatment, after which they decreased spontaneously and disappeared by the 21st day. Therefore, a bone marrow examination was not performed at that time.

On the 22nd day of treatment, eltrombopag 75 mg/day once daily was started, which was initially scheduled to be administered on the 15th day of treatment but was postponed due to the appearance of plasma cells. After that, there was no recurrence of plasma cell proliferation, and an increasing trend in neutrophils was observed from day 45 of treatment. At three months, the patient achieved a partial response with leukocytes 3600/μL (neutrophils 2358/μL), Hb 8.7 g/dL (reticulocytes 69120/μL), and platelets 85,000/μL, all in the transfusion-independent setting. At the six-month follow-up, she achieved a complete response with leukocytes 4700/μL (neutrophils 3055/μL), Hb 10.3 g/dL (reticulocytes 76560/μL), and platelets 111,000/μL. She continues to be treated with cyclosporine and eltrombopag in an outpatient setting.

## Discussion

**Figure 4:**
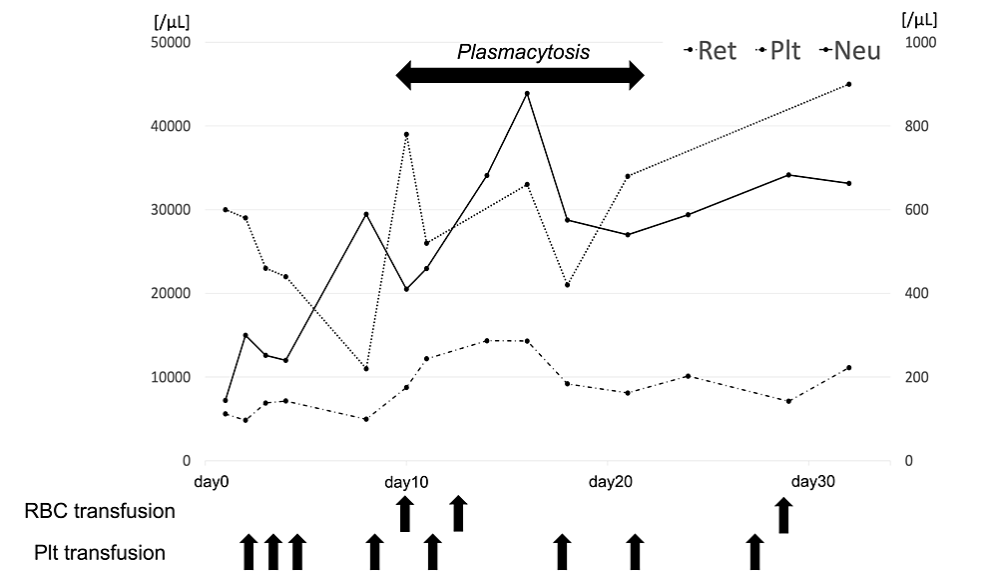
Plasmacytosis and hematopoietic recovery in peripheral blood Consistent with the timing of plasmacytosis, there has been a recovery of neutrophils. Additionally, there has been a decrease in the frequency of RBC and Plt transfusions. The left vertical axis indicates the values of reticulocytes and platelets, and the right vertical axis indicates the values of neutrophils. μL: microliter; Ret: reticulocyte; PLT: platelet; Neu: neutrophil

In this case, an increase in reactive plasma cells was observed in the peripheral blood immediately after immunosuppressive therapy with ATG and cyclosporine for aplastic anemia. Previous reports have shown reactive plasma cells in patients with aplastic anemia after infection and other reasons [5,6]. However, to our knowledge, this is the first report of reactive plasma cells in peripheral blood immediately after immunosuppressive therapy for aplastic anemia. This is true for both horse and rabbit ATG. The appearance of plasma cells in the peripheral blood can be caused by reactive factors such as infection, autoimmune diseases, and non-hematologic malignant tumors [7]. However, it is crucial first to identify neoplastic factors, as we did in this case. There have been reports of co-existing multiple myeloma at the time of diagnosis or later in aplastic anemia, although it is very rare [8,9]. There have been reports of developing multiple myeloma after immunosuppressive therapy [10,11]. However, this case is unique in the time course that the plasma cells appeared immediately after immunosuppressive therapy. Flow cytometry and other tests showed no clonality of plasma cells in this case, indicating reactive plasma cells. We believe that immunosuppressive therapy with ATG reactively mobilized plasma cells in the peripheral blood. In particular, this finding may reflect the recovery of hematopoiesis. Figure 4 shows a recovery trend of neutrophils following the timing of the detection of plasma cells in the peripheral blood. The patient's hematopoietic recovery remained good, and a complete response was achieved six months after the start of treatment. Therefore, the appearance of plasma cells in the peripheral blood, although rare, preceded the subsequent good recovery of hematopoietic function.

## Conclusions

We report a case of increased reactive plasma cells in peripheral blood immediately following immunosuppressive therapy with ATG and cyclosporine for aplastic anemia. Plasmacytosis can occur due to a variety of reasons, including infections, autoimmune diseases and neoplastic disorders. Thus, when a plasmacytosis is observed, it is essential to first distinguish tumorigenesis by morphology and flow cytometry. In this case, the increased plasma cells indicated a good hematopoietic response to immunosuppressive therapy.

## References

[1] Young NS (2018). Aplastic anemia. N Engl J Med.

[2] Peffault de Latour R, Kulasekararaj A, Iacobelli S (2022). Eltrombopag added to immunosuppression in severe aplastic anemia. N Engl J Med.

[3] Young N, Griffith P, Brittain E (1988). A multicenter trial of antithymocyte globulin in aplastic anemia and related diseases. Blood.

[4] Scheinberg P, Nunez O, Weinstein B, Scheinberg P, Biancotto A, Wu CO, Young NS (2011). Horse versus rabbit antithymocyte globulin in acquired aplastic anemia. N Engl J Med.

[5] Nishimoto Y, Iwahashi T, Nishihara T, Katayama H, Kuribayashi K, Takao T, Saito K (1987). Hepatitis-associated aplastic anemia with systemic plasmacytosis. Acta Pathol Jpn.

[6] Yamamoto M, Kumekawa H, Murata Y (2012). Marked reactive plasmacytosis accompanied by drug eruption in a patient with aplastic anemia [Article in Japanese]. Rinsho Ketsueki.

[7] Pellat-Deceunynck C, Jego G, Robillard N, Accard F, Amiot M, Bataille R (2000). Reactive plasmacytoses, a model for studying the biology of human plasma cell progenitors and precursors. Hematol J.

[8] Manoharan A, Horsley R, Pitney WR (1981). Myelomatosis in aplastic anaemia--a true association or fortuitous occurrence?. Pathology.

[9] Medhi K, Kalita D, Chopra A, Anand M, Raina V, Kumar R (2008). Multiple myeloma presenting with coexisting severe marrow hypoplasia. Indian J Pathol Microbiol.

[10] Beyer GS, Glant MD, Hoffman R (1986). Development of IgA multiple myeloma in a patient with aplastic anemia treated with antithymocyte globulin. N Engl J Med.

[11] Murray NP, Ruiz MA, Miranda GM (2017). CD19 ((+)) CD56 ((-)) myeloma arising in a patient who failed two courses of immunosupressive therapy for aplastic anaemia. Ecancermedicalscience.

